# Editorial: Novel therapeutic targets for lower urinary tract symptoms

**DOI:** 10.3389/fruro.2023.1239287

**Published:** 2023-06-26

**Authors:** Jianshu Ni, Baojun Gu, Naoki Yoshimura

**Affiliations:** ^1^ Department of Urology, Shanghai Ninth People's Hospital, Shanghai Jiao Tong University School of Medicine, Shanghai, China; ^2^ Department of Urology, Shanghai Sixth People's Hospital, Shanghai Jiao Tong University, Shanghai, China; ^3^ Department of Urology, University of Pittsburgh School of Medicine, Pittsburgh, PA, United States

**Keywords:** lower urinary tract symptom (LUTS), therapeutic target, psychological stress, bladder outlet obstruction, neurotrophin

In our Research Topic, several high-quality papers were collected focusing on different fields including interstitial cystitis, stress-induced voiding dysfunction and bladder outlet obstruction. Here we would like to highlight the following works.

Interstitial cystitis/bladder pain syndrome(IC/BPS) is a debilitating chronic disease of unknown etiology characterized by lower urinary tract symptoms such as increased urinary urgency and frequency, bladder discomfort, decreased bladder capacity, and pelvic pain. Although many therapeutic options are available for patients with IC/BPS, there are a limited number of effective treatments. Thus, basic and translational studies are anticipated to explore the specific mechanisms and novel therapy targets. Whitman et al. summarized the potential relationship between small fiber polyneuropathy (SFPN) and chronic pain disorders including IC/BPS, and they propose that routine SFPN testing should be included in the clinical work-up of patients with IC/BPS who are refractory to multiple treatments or have multiple comorbid pain syndromes to further elucidate their relationship. Hsiang et al. focused on nerve growth factor(NGF) signaling in the IC/BPS from the basic research aspect. They demonstrated that pharmacological inhibition of tTrkA, TrkB and p75NTR receptors could improve bladder function (increased intermicturition interval and bladder capacity) in a mouse model of cyclophosphamide (CYP)-induced cystitis, indicating these neurotrophin receptors may be potential therapeutic targets for IC/BPS and other inflammatory disorders of the bladder. Furthermore, they explored the effects of CYP treatment and pharmacological inhibition of p75NTR and TrkA on NGF signaling–related proteins: NGF, TrkA, phosphorylated (p)-TrkA, p75NTR, p-ERK1/2, and p-JNK. The results showed that increased urothelial NGF expression, decreased TrkA and p75NTR expression, as well as altered TrkA/p75NTR ratio, phosphorylation of ERK1/2 and JNK under the cystitis condition. Additionally, both TrkA and p75NTR inhibition affected the activation of downstream signaling pathways of TrkA, supporting the hypothesis that NGF actions during cystitis are primarily TrkA-mediated.

Psychological stress is associated with lower urinary tract (LUT) symptom (e.g., increased voiding frequency, urgency and pelvic pain) onset and aggravation; however, the mechanisms underlying the effects of stress on urinary bladder function and pelvic pain are not well elucidated. Sidwell et al. used a female mice model of repeated variate stress (RVS) and examined the bladder function, anxiety-like behavior, and TRPV transcript expression in the urinary bladder and lumbosacral spinal cord and associated dorsal root ganglia (DRG) at 2 week (wk) or 4 wk duration. The results demonstrated significant changes in TrpV1 and TrpV4 mRNA expression between control and RVS cohorts in the urothelium, lumbosacral spinal cord, and DRG, which might be future directions for the treatment of stress-related bladder dysfunction. Also, Girard et al. established a novel stress-induced symptom exacerbation (SISE) mouse model, combining cyclophosphamide (CYP) administration with repeated variate stress (RVS), which demonstrates increased urinary frequency and somatic (pelvic and hindpaw) sensitivity. Besides, CYP+RVS produced the largest increase in inflammatory mediators (NGF, BDNF, CXC and IL-6) in the urinary bladder compared to CYP alone or RVS alone. The SISE model of CYP+RVS will be useful to address underlying mechanisms where psychological stress exacerbates symptoms in functional bladder disorders leading to identification of targets and potential treatments.

Benign prostatic hyperplasia (BPH) and bladder outlet obstruction (BOO) patients can both have voiding and storage symptoms. While the voiding symptoms can be solved by surgery to relieve the obstruction, the storage symptoms associated with detrusor overactivity (DO) can be persistent in 20-40% of patients. The underlying mechanisms of the association between DO/LUTS and the changes in the bladder occurring after relief of BOO have not been clarified. Andersson and Uvelius summarized the electron microscopic results and microarray analysis from obstructed and de-obstructed bladder tissues to identify the structural and molecular changes. They drew the conclusion that although voiding function is rapidly normalized after release of outflow obstruction and many of the morphological changes are reversed, the de-obstructed rat bladder has irreversible gene expressions distinctly different from both control and obstructed bladders. Further studies are worthwhile to re-assess the development potential for e.g., endothelin receptor antagonists, purinergic receptor antagonists and Rho-kinase inhibitors.

## Author contributions

All authors listed have made a substantial, direct, and intellectual contribution to the work and approved it for publication.

